# Supplementary material for: In Vitro Salivary Protein Adsorption Profile on Titanium and Ceramic Surfaces and the Corresponding Putative Immunological Implications
Source: Int J Mol Sci. 2020 Apr 27;21(9):3083. doi: 10.3390/ijms21093083 (PMC7247707; doi:10.3390/ijms21093083)
Supplement: Supplementary file 1 [file ijms-21-03083-s001.pdf]

Supplementary material

Table S1. Acquired salivary proteins and peptides detected on titanium and feldspathic ceramic surfaces

Accession ID	Protein	Gene	Mol. weight [kDa]	Q-value[a]	Score[b]	Titanium	Ceramic
P61604	10 kDa heat shock protein	*HSPE1*	10.932	0	38.331	+	+
P31946	14-3-3 protein beta/alpha	*YWHAB*	28.082	0	27.442	-	+
P62258	14-3-3 protein epsilon	*YWHAE*	29.174	0	26.999	+	+
P31947	14-3-3 protein sigma	*SFN*	27.774	0	323.310	+	+
P27348	14-3-3 protein theta	*YWHAQ*	27.764	0	6.026	+	+
P63104	14-3-3 protein zeta/delta	*YWHAZ*	27.745	0	323.310	+	+
E5RGC7	28S ribosomal protein S28	*MRPS28*	11.747	0.001	2.110	+	-
P46783	40S ribosomal protein S10	*RPS10*	18.898	0	7.188	+	-
P25398	40S ribosomal protein S12	*RPS12*	14.515	0	12.302	+	+
I3L3P7	40S ribosomal protein S15a	*RPS15A*	11.477	0	5.457	+	-
P62269	40S ribosomal protein S18	*RPS18*	17.718	0	44.435	+	-
H0YEN5	40S ribosomal protein S2	*RPS2*	21.154	0	10.343	+	-
H0Y8L7	40S ribosomal protein S3a	*RPS3A*	22.137	0.002	3.664	+	-
P62701	40S ribosomal protein S4, X isoform	*RPS4X*	29.597	0	32.952	+	-
P62753	40S ribosomal protein S6	*RPS6*	28.680	0	7.289	+	-
P62081	40S ribosomal protein S7	*RPS7*	22.127	0	12.799	+	-
Q5JR95	40S ribosomal protein S8	*RPS8*	21.879	0	21.677	+	-
P10809	60 kDa heat shock protein	*HSPD1*	61.054	0	184.440	+	+
P05386	60S acidic ribosomal protein P1	*RPLP1*	11.514	0	25.891	+	+
P05387	60S acidic ribosomal protein P2	*RPLP2*	11.665	0	35.789	+	-
P62906	60S ribosomal protein L10a	*RPL10A*	24.831	0.001	1.912	+	-
P30050	60S ribosomal protein L12	*RPL12*	17.818	0	36.473	+	-

Accession ID	Protein	Gene	Mol. weight [kDa]	Q-value[a]	Score[b]	Titanium	Ceramic
P26373	60S ribosomal protein L13	*RPL13*	24.261	0	9.378	+	-
J3QQT2	60S ribosomal protein L17	*RPL17*	19.586	0	40.597	+	-
P46778	60S ribosomal protein L21	*RPL21*	18.565	0	26.934	+	-
E9PJD9	60S ribosomal protein L27a	*RPL27A*	10.127	0.001	2.704	+	-
J3KT73	60S ribosomal protein L38	*RPL38*	7.5649	0	90.283	+	-
Q02878	60S ribosomal protein L6	*RPL6*	32.728	0	27.736	+	+
E9PKZ0	60S ribosomal protein L8	*RPL8*	22.389	0.002	3.316	+	-
P52209	6-phosphogluconate dehydrogenase	*PGD*	53.139	0	154.670	+	+
P11021	78 kDa glucose-regulated protein	*HSPA5*	72.332	0	159.160	+	+
H0YN26	Acidic leucine-rich nuclear phosphoprotein 32 family member A	*ANP32A*	19.997	0	9.414	+	-
A2A274	Aconitate hydratase	*ACO2*	87.819	0	73.748	-	+
P68133	Actin, alpha skeletal muscle	*ACTA1*	42.051	0	61.940	+	+
P60709	Actin, cytoplasmic 1	*ACTB*	41.736	0	323.310	+	+
P63261	Actin, cytoplasmic 2	*ACTG1*	41.792	0	149.770	+	+
F8VR50	Actin-related protein 2/3 complex subunit 3	*ARPC3*	9.7261	0	5.957	+	-
F8WCF6	Actin-related protein 2/3 complex subunit 4	*ARPC4*	21.058	0	6.376	-	+
B8ZWD1	Acyl-CoA-binding protein	*DBI*	11.150	0	49.419	+	+
P23526	Adenosylhomocysteinase	*AHCY*	47.716	0	31.345	+	-
F8W1A4	Adenylate kinase 2	*AK2*	25.630	0	13.275	+	-
Q01518	Adenylyl cyclase-associated protein 1	*CAP1*	51.901	0	66.387	+	+
Q15847	Adipogenesis regulatory factor	*ADIRF*	7.855	0	28.569	+	-
F5H423	ADP-ribosylation factor 1	*ARF1*	23.346	0.002	3.866	+	-
C9JMC5	Aldehyde dehydrogenase, dimeric NADP-preferring	*ALDH3A1*	41.647	0	43.332	+	+
P02763	Alpha-1-acid glycoprotein 1	*ORM1*	23.511	0	99.424	+	+
P01011	Alpha-1-antichymotrypsin	*SERPINA3*	47.650	0.002	4.147	+	-
P01009	Alpha-1-antitrypsin	*SERPINA1*	46.736	0	323.310	+	+

Accession ID	Protein	Gene	Mol. weight [kDa]	Q-value[a]	Score[b]	Titanium	Ceramic
P04217	Alpha-1B-glycoprotein	*A1BG*	54.253	0	20.495	+	-
C9JV77	Alpha-2-HS-glycoprotein	*AHSG*	39.411	0	323.310	+	+
P01023	Alpha-2-macroglobulin	*A2M*	163.290	0	323.310	+	+
A8K2U0	Alpha-2-macroglobulin-like protein 1	*A2ML1*	161.100	0	139.270	+	+
O43707	Alpha-actinin-4	*ACTN4*	104.850	0	45.992	+	+
P04745	Alpha-amylase 1	*AMY1A*	57.767	0	323.310	+	+
P06733	Alpha-enolase	*ENO1*	47.168	0	323.310	+	+
H7C0V9	Amyloid beta A4 protein	*APP*	54.981	0	11.414	+	+
P03950	Angiogenin	*ANG*	16.550	0	14.935	+	+
P04083	Annexin A1	*ANXA1*	38.714	0	274.520	+	+
P07355	Annexin A2	*ANXA2*	38.604	0	204.250	+	+
P03973	Antileukoproteinase	*SLPI*	14.326	0	323.310	+	+
P01008	Antithrombin-III	*SERPINC1*	52.602	0	9.259	+	-
P02647	Apolipoprotein A-I	*APOA1*	30.777	0	323.310	+	+
V9GYG9	Apolipoprotein A-II	*APOA2*	10.649	0	52.050	+	+
P06727	Apolipoprotein A-IV	*APOA4*	45.398	0	172.230	+	+
P04114	Apolipoprotein B-100	*APOB*	489.830	0	16.214	-	+
P54253	Ataxin-1	*ATXN1*	86.922	0.001	2.986	-	+
P25705	ATP synthase subunit alpha	*ATP5A1*	59.750	0	123.550	-	+
P06576	ATP synthase subunit beta	*ATP5B*	56.559	0	223.450	+	-
O15523	ATP-dependent RNA helicase DDX3Y	*DDX3Y*	73.153	0	8.269	+	-
G3V1R1	Basic salivary proline-rich protein 1	*PRB1*	17.672	0	6.693	+	+
P02812	Basic salivary proline-rich protein 2	*PRB2*	40.799	0	171.050	+	+
F5H7C1	Basic salivary proline-rich protein 3	*PRB3*	35.122	0	17.983	+	+
E7EXA8	Basic salivary proline-rich protein 4	*PRB4*	18.011	0	24.669	+	+
P02749	Beta-2-glycoprotein 1	*APOH*	38.298	0	160.400	+	+

Accession ID	Protein	Gene	Mol. weight [kDa]	Q-value[a]	Score[b]	Titanium	Ceramic
H0YL18	Beta-2-microglobulin	*B2M*	13.972	0	122.650	+	+
P07686	Beta-hexosaminidase subunit beta	*HEXB*	63.111	0	6.542	-	+
Q9NP55	BPI fold-containing family A member 1	*BPIFA1*	26.712	0	6.238	+	-
Q96DR5	BPI fold-containing family A member 2	*BPIFA2*	27.011	0	227.990	+	+
Q8TDL5	BPI fold-containing family B member 1	*BPIFB1*	52.441	0	154.920	+	+
Q8N4F0	BPI fold-containing family B member 2	*BPIFB2*	49.172	0	295.060	+	+
P80723	Brain acid soluble protein 1	*BASP1*	22.693	0	6.496	+	+
H0Y7A7	Calmodulin	*CALM1*	20.762	0	141.550	+	+
P27482	Calmodulin-like protein 3	*CALML3*	16.891	0	240.510	+	+
Q9NZT1	Calmodulin-like protein 5	*CALML5*	15.892	0	18.248	+	+
P27797	Calreticulin	*CALR*	48.141	0	144.050	+	+
O43852	Calumenin	*CALU*	37.106	0	7.479	+	+
E5RH81	Carbonic anhydrase 1	*CA1*	19.236	0	12.570	+	-
P23280	Carbonic anhydrase 6	*CA6*	35.366	0	323.310	+	+
P07858	Cathepsin B	*CTSB*	37.821	0	114.650	+	+
P07339	Cathepsin D	*CTSD*	44.552	0	49.403	+	+
Q5T8F0	Cathepsin L1	*CTSL*	25.500	0.006	1.194	+	-
P60953	Cell division control protein 42 homolog	*CDC42*	21.258	0	5.398	+	-
P29373	Cellular retinoic acid-binding protein 2	*CRABP2*	15.693	0	7.859	+	-
P00450	Ceruloplasmin	*CP*	122.200	0	77.473	+	+
P36222	Chitinase-3-like protein 1	*CHI3L1*	42.625	0.001	2.668	-	+
P10909	Clusterin	*CLU*	52.494	0	138.440	+	+
P00748	Coagulation factor XII	*F12*	67.791	0	74.668	+	+
P23528	Cofilin-1	*CFL1*	18.502	0	208.150	+	+
Q96CT7	Coiled-coil domain-containing protein 124	*CCDC124*	25.835	0.005	1.317	+	-
P02452	Collagen alpha-1(I) chain	*COL1A1*	138.940	0	84.482	+	+

Accession ID	Protein	Gene	Mol. weight [kDa]	Q-value[a]	Score[b]	Titanium	Ceramic
P08123	Collagen alpha-2(I) chain	*COL1A2*	129.150	0	119.690	+	+
P01024	Complement C3	*C3*	187.150	0	323.310	+	+
P0C0L4	Complement C4-B	*C4B*	192.780	0	287.410	+	+
Q07021	Complement component 1 Q subcomponent-binding protein	*C1QBP*	31.362	0	50.237	+	-
B4E1Z4	Complement factor B	*CFB*	140.940	0	66.899	+	+
P08603	Complement factor H	*CFH*	139.090	0	253.340	+	+
P35321	Cornifin-A	*SPRR1A*	9.877	0	107.850	+	+
P22528	Cornifin-B	*SPRR1B*	9.888	0	158.830	+	+
Q9UBG3	Cornulin	*CRNN*	53.533	0	323.310	+	+
P31146	Coronin-1A;Coronin	*CORO1A*	51.026	0	90.289	+	+
P04080	Cystatin-B	*CSTB*	11.139	0	134.160	+	+
P01034	Cystatin-C	*CST3*	15.799	0	226.520	+	+
P28325	Cystatin-D	*CST5*	16.080	0	323.310	+	+
P01036	Cystatin-S	*CST4*	16.214	0	323.310	+	+
P09228	Cystatin-SA	*CST2*	16.445	0	289.390	+	+
P01037	Cystatin-SN	*CST1*	16.387	0	323.310	+	+
J3KPA1	Cysteine-rich secretory protein 3	*CRISP3*	30.975	0	173.210	+	+
P32320	Cytidine deaminase	*CDA*	16.185	0	20.168	+	+
Q9UGM3	Deleted in malignant brain tumors 1 protein	*DMBT1*	260.730	0	323.310	+	+
Q02487	Desmocollin-2	*DSC2*	99.961	0	155.040	+	+
Q02413	Desmoglein-1	*DSG1*	113.750	0.002	3.501	+	+
P32926	Desmoglein-3	*DSG3*	107.530	0	63.865	+	+
F6RFD5	Destrin	*DSTN*	15.397	0.002	3.231	+	-
O60216	Double-strand-break repair protein rad21	*RAD21*	71.689	0.003	1.707	+	-
Q76N89	E3 ubiquitin-protein ligase HECW1	*HECW1*	179.550	0.003	1.704	+	+
Q12805	EGF-containing fibulin-like extracellular matrix protein 1	*EFEMP1*	54.640	0	39.828	+	+

Accession ID	Protein	Gene	Mol. weight [kDa]	Q-value[a]	Score[b]	Titanium	Ceramic
P19957	Elafin	*PI3*	12.269	0	8.603	+	+
Q5VTE0	Elongation factor 1-alpha 1	*EEF1A1*	50.184	0	92.366	+	+
E9PNW6	Elongation factor 1-delta	*EEF1D*	4.227	0.001	3.029	+	-
P26641	Elongation factor 1-gamma	*EEF1G*	50.118	0	26.082	+	+
P13639	Elongation factor 2	*EEF2*	95.337	0	64.550	+	+
P14625	Endoplasmin	*HSP90B1*	92.468	0	28.754	+	-
Q5HYB6	Epididymis luminal protein 189	*TPM3*	27.175	0	51.025	+	+
Q16610	Extracellular matrix protein 1	*ECM1*	60.673	0.002	4.097	+	+
E7EQR4	Ezrin	*EZR*	69.371	0	225.510	+	+
Q01469	Fatty acid-binding protein	*FABP5*	15.164	0	323.310	+	+
P02671	Fibrinogen alpha chain	*FGA*	94.972	0	323.310	+	+
P02675	Fibrinogen beta chain	*FGB*	55.928	0	323.310	+	+
C9JEU5	Fibrinogen gamma chain	*FGG*	50.322	0	228.070	+	+
P02751	Fibronectin	*FN1*	262.620	0	105.410	+	+
P23142	Fibulin-1	*FBLN1*	77.213	0.001	3.110	+	-
Q5HY54	Filamin-A	*FLNA*	276.550	0	10.279	+	-
P15328	Folate receptor alpha	*FOLR1*	29.819	0	171.500	+	+
Q8NFU4	Follicular dendritic cell secreted peptide	*FDCSP*	9.700	0.001	2.101	+	+
P04075	Fructose-bisphosphate aldolase A	*ALDOA*	39.420	0	200.800	+	+
P09972	Fructose-bisphosphate aldolase C	*ALDOC*	39.455	0	19.610	+	+
P09382	Galectin-1	*LGALS1*	14.716	0	32.436	+	-
P17931	Galectin-3	*LGALS3*	26.152	0	6.677	+	-
Q08380	Galectin-3-binding protein	*LGALS3BP*	65.330	0	205.070	+	+
P06744	Glucose-6-phosphate isomerase	*GPI*	64.824	0	57.487	+	+
P14314	Glucosidase 2 subunit beta	*PRKCSH*	60.192	0	84.794	+	+
P09211	Glutathione S-transferase P	*GSTP1*	23.356	0	323.310	+	+

Accession ID	Protein	Gene	Mol. weight [kDa]	Q-value[a]	Score[b]	Titanium	Ceramic
P04406	Glyceraldehyde-3-phosphate dehydrogenase	*GAPDH*	36.053	0	323.310	+	+
Q92896	Golgi apparatus protein 1	*GLG1*	134.550	0	46.042	+	+
Q8NBJ4	Golgi membrane protein 1	*GOLM1*	45.333	0	6.801	+	+
P28799	Granulins	*GRN*	63.544	0	145.560	+	+
Q9HAV7	GrpE protein homolog 1	*GRPEL1*	24.279	0.001	2.876	+	-
B5MDF5	GTP-binding nuclear protein Ran	*RAN*	26.224	0	13.673	+	+
P00738	Haptoglobin	*HP*	45.205	0	297.690	+	+
P08107	Heat shock 70 kDa protein 1A/1B	*HSPA1A*	70.051	0	105.400	+	+
P17066	Heat shock 70 kDa protein 6	*HSPA6*	71.027	0	10.188	+	+
E9PKE3	Heat shock cognate 71 kDa protein	*HSPA8*	68.805	0	105.970	+	+
O75506	Heat shock factor-binding protein 1	*HSBP1*	8.5435	0	10.867	-	+
P04792	Heat shock protein beta-1	*HSPB1*	22.782	0	11.232	+	+
P07900	Heat shock protein HSP 90-alpha	*HSP90AA1*	84.659	0	186.580	+	+
P08238	Heat shock protein HSP 90-beta	*HSP90AB1*	83.263	0	161.300	+	+
Q8NDA2	Hemicentin-2	*HMCN2*	62.860	0.005	1.290	+	+
P69905	Hemoglobin subunit alpha	*HBA1*	15.257	0	323.310	+	+
P68871	Hemoglobin subunit beta	*HBB*	15.998	0	323.310	+	+
P02042	Hemoglobin subunit delta	*HBD*	16.055	0	35.870	+	+
P02790	Hemopexin	*HPX*	51.676	0	215.130	+	+
P51858	Hepatoma-derived growth factor	*HDGF*	26.788	0	157.130	+	+
D6R9P3	Heterogeneous nuclear ribonucleoprotein A/B	*HNRNPAB*	30.302	0	15.992	+	-
D6RD83	Heterogeneous nuclear ribonucleoprotein D0	*HNRNPD*	9.9407	0.004	1.516	+	-
Q5T6W5	Heterogeneous nuclear ribonucleoprotein K	*HNRNPK*	47.557	0	29.681	+	-
G3V576	Heterogeneous nuclear ribonucleoproteins C1/C2	*HNRNPC*	25.256	0	24.166	+	+
P05204	High mobility group nucleosome-binding domain-containing protein 3	*HMGN3*	9.393	0.005	1.433	+	+

Accession ID	Protein	Gene	Mol. weight [kDa]	Q-value[a]	Score[b]	Titanium	Ceramic
P09429	High mobility group protein B1	*HMGB1*	24.893	0	60.708	+	+
P26583	High mobility group protein B2	*HMGB2*	24.033	0	93.792	+	+
P15515	Histatin-1;His1-(31-57)-peptide	*HTN1*	6.963	0	191.070	+	+
P04196	Histidine-rich glycoprotein	*HRG*	59.578	0	194.230	+	+
P16403	Histone H1.2	*HIST1H1C*	21.364	0	12.666	+	-
P16401	Histone H1.5	*HIST1H1B*	22.580	0.002	3.647	+	-
Q16777	Histone H2A type 2-C	*HIST2H2AC*	13.988	0	102.700	+	+
Q71UI9	Histone H2A.V	*H2AFV*	13.509	0	71.539	+	-
U3KQK0	Histone H2B type 1-K	*HIST1H2BK*	18.804	0	50.412	+	+
Q5TEC6	Histone H3	*HIST2H3PS2*	15.430	0	7.789	+	-
Q71DI3	Histone H3.2	*HIST2H3A*	15.388	0	21.176	+	+
P62805	Histone H4	*HIST1H4A*	11.367	0	258.180	+	+
H7C3I1	Hsc70-interacting protein	*ST13*	16.219	0.002	4.758	+	+
P01876	Ig alpha-1 chain C region	*IGHA1*	37.654	0	323.310	+	+
A0A075B6N7	Ig alpha-2 chain C region	*IGHA2*	36.591	0	193.910	+	+
P01877	Ig alpha-2 chain C region	*IGHA2*	36.526	0	42.274	+	+
A0A087WV47	Ig gamma-1 chain C region	*IGHG1*	51.153	0	323.310	+	+
A0A087X079	Ig gamma-1 chain C region	*IGHG1*	51.912	0.001	1.868	-	+
A0A087WYC5	Ig gamma-1 chain C region	*IGHG1*	52.426	0	4.955	+	+
P01859	Ig gamma-2 chain C region	*IGHG2*	35.900	0	139.770	+	+
A0A087WXL8	Ig gamma-3 chain C region	*IGHG3*	56.889	0	174.090	+	+
P01861	Ig gamma-4 chain C region	*IGHG4*	35.940	0	85.277	+	+
P01766	Ig heavy chain V-III region BRO	*IGHV3-13*	13.227	0	112.740	+	+
P01765	Ig heavy chain V-III region TIL	*IGHV3-23*	11.612	0	118.390	+	+
P01598	Ig kappa chain V-I region EU	*IGKV1-5*	11.788	0	81.106	+	+
A0A087X0N5	Ig kappa chain V-I region WAT	*IGKV1D-33*	10.494	0	94.219	+	+

Accession ID	Protein	Gene	Mol. weight [kDa]	Q-value[a]	Score[b]	Titanium	Ceramic
P01611	Ig kappa chain V-I region Wes	*IGKV1D-12*	11.608	0	5.528	+	+
P06310	Ig kappa chain V-II region Cum	*IGKV2D-29*	11.192	0	14.854	+	+
P01621	Ig kappa chain V-III region NG9	*IGKV3-20*	10.729	0.003	1.535	+	+
P01623	Ig kappa chain V-III region WOL	*IGKV3-20*	11.746	0	92.691	+	+
P01625	Ig kappa chain V-IV region Len	*IGKV4-1*	12.640	0	115.510	+	+
P01702	Ig lambda chain V-I region BL2	*IGLV1-51*	12.619	0.005	1.414	+	+
P04208	Ig lambda chain V-I region WAH	*IGLV1-47*	12.283	0	5.437	+	+
P80748	Ig lambda chain V-III region LOI	*IGLV3-9*	11.935	0	83.806	+	+
P01714	Ig lambda chain V-III region SH	*IGLV3-19*	11.392	0	38.193	+	+
P01717	Ig lambda chain V-IV region Hil	*IGLV3-25*	11.517	0	49.877	+	+
P0CG06	Ig lambda-2 chain C regions/Ig lambda-3 chain C regions	*IGLC2/IGLC3*	11.208	0	228.480	+	+
A0A087X2C0	Ig mu chain C region	*IGHM*	64.106	0	323.310	+	+
A0A087WYJ9	Ig mu heavy chain disease protein	*IGHM*	65.700	0.001	2.960	+	+
Q9Y6R7	IgGFc-binding protein	*FCGBP*	445.210	0	65.358	+	+
Q9GZP8	Immortalization up-regulated protein	*IMUP*	10.897	0.001	2.773	+	+
P01591	Immunoglobulin J chain	*IGJ*	18.098	0	77.552	+	+
P0CG04	Immunoglobulin lambda-like polypeptide 5	*IGLL5, IGLC1*	24.683	0	17.173	+	+
Q16270	Insulin-like growth factor-binding protein 7	*IGFBP7*	29.130	0.001	2.873	+	+
P19827	Inter-alpha-trypsin inhibitor heavy chain H1	*ITIH1*	101.390	0	7.165	+	-
Q5T985	Inter-alpha-trypsin inhibitor heavy chain H2	*ITIH2*	105.210	0	120.940	+	+
B7ZKJ8	Inter-alpha-trypsin inhibitor heavy chain H4	*ITIH4*	103.880	0.002	4.067	+	-
P18510	Interleukin-1 receptor antagonist protein	*IL1RN*	20.055	0	50.089	+	+
Q9UHA7	Interleukin-36 alpha	*IL36A*	17.684	0.002	4.471	+	-
P07476	Involucrin	*IVL*	68.478	0	12.321	+	-
P06870	Kallikrein-1	*KLK1*	28.889	0	96.828	+	+
J3KNA1	Kinesin heavy chain isoform 5C	*KIF5C*	107.250	0.005	1.278	+	-

Accession ID	Protein	Gene	Mol. weight [kDa]	Q-value[a]	Score[b]	Titanium	Ceramic
P01042	Kininogen-1	*KNG1*	71.957	0	85.087	+	+
F5H386	Lactoperoxidase	*LPO*	73.956	0	323.310	+	+
P02788	Lactotransferrin	*LTF*	78.181	0	323.310	+	+
P02545	Lamin-A/C	*LMNA*	74.139	0	6.349	+	+
P30740	Leukocyte elastase inhibitor	*SERPINB1*	42.741	0	32.787	+	+
P31025	Lipocalin-1	*LCN1*	19.250	0	179.750	+	+
A8MW50	L-lactate dehydrogenase	*LDHB*	25.218	0	16.498	+	-
P00338	L-lactate dehydrogenase A chain	*LDHA*	36.688	0	31.699	+	+
Q9BZG9	Ly-6/neurotoxin-like protein 1	*LYNX1*	14.026	0	27.554	+	-
P61626	Lysozyme C	*LYZ*	16.537	0	323.310	+	+
P14174	Macrophage migration inhibitory factor	*MIF*	12.476	0	15.449	+	+
P40926	Malate dehydrogenase	*MDH2*	35.503	0	57.354	-	+
P08493	Matrix Gla protein	*MGP*	12.353	0	63.739	+	+
P14780	Matrix metalloproteinase-9	*MMP9*	78.457	0	46.737	+	+
P55145	Mesencephalic astrocyte-derived neurotrophic factor	*MANF*	20.700	0	21.957	+	+
Q5H9A7	Metalloproteinase inhibitor 1	*TIMP1*	16.057	0	124.980	+	+
P26038	Moesin	*MSN*	67.819	0	87.902	+	+
D6RFL4	Monocyte differentiation antigen CD14	*CD14*	23.313	0.001	2.378	+	-
Q9HC84	Mucin-5B	*MUC5B*	596.330	0	323.310	+	+
Q8TAX7	Mucin-7	*MUC7*	39.158	0	67.663	+	+
U3KPS2	Myeloblastin	*PRTN3*	23.611	0	7.119	+	+
P05164	Myeloperoxidase	*MPO*	83.868	0	160.600	+	+
F8W1R7	Myosin light polypeptide 6	*MYL6*	16.290	0	23.955	+	+
J3QRS3	Myosin regulatory light chain 12A	*MYL12A*	20.457	0	17.315	+	-
P35579	Myosin-9	*MYH9*	226.530	0	164.400	+	+
F8W1N5	Nascent polypeptide-associated complex subunit alpha	*NACA*	7.813	0	43.602	+	+

Accession ID	Protein	Gene	Mol. weight [kDa]	Q-value[a]	Score[b]	Titanium	Ceramic
Q09666	Neuroblast differentiation-associated protein AHNAK	*AHNAK*	629.090	0	4.875	+	+
P59666	Neutrophil defensin 3	*DEFA3*	10.245	0	39.708	+	+
P08246	Neutrophil elastase	*ELANE*	28.518	0	13.057	+	+
X6R8F3	Neutrophil gelatinase-associated lipocalin	*LCN2*	22.788	0	104.720	+	+
P43490	Nicotinamide phosphoribosyltransferase	*NAMPT*	55.520	0	14.930	+	+
P10153	Non-secretory ribonuclease	*RNASE2*	18.354	0.002	3.685	+	+
Q02818	Nucleobindin-1	*NUCB1*	53.879	0	153.450	+	+
V9HW75	Nucleobindin-2	*NUCB2*	50.222	0	323.310	+	+
P19338	Nucleolin	*NCL*	76.613	0	64.895	+	+
P06748	Nucleophosmin	*NPM1*	32.575	0	186.160	+	+
Q32Q12	Nucleoside diphosphate kinase	*NME1, NME2*	32.642	0	16.465	-	+
Q92882	Osteoclast-stimulating factor 1	*OSTF1*	23.787	0.002	4.013	+	+
P20962	Parathymosin	*PTMS*	11.530	0.001	2.112	+	+
O75475	PC4 and SFRS1-interacting protein	*PSIP1*	60.103	0.002	4.468	+	-
P19021	Peptidyl-glycine alpha-amidating monooxygenase	*PAM*	108.330	0	22.469	+	-
P62937	Peptidyl-prolyl cis-trans isomerase A	*PPIA*	18.012	0	164.450	+	+
P23284	Peptidyl-prolyl cis-trans isomerase B	*PPIB*	23.742	0	210.760	+	+
P62942	Peptidyl-prolyl cis-trans isomerase FKBP1A	*FKBP1A*	14.926	0	18.777	+	-
Q06830	Peroxiredoxin-1	*PRDX1*	22.110	0	28.721	+	+
P32119	Peroxiredoxin-2	*PRDX2*	21.892	0.001	3.052	+	-
P30044	Peroxiredoxin-5	*PRDX5*	22.086	0	33.396	+	+
P30041	Peroxiredoxin-6	*PRDX6*	25.035	0	19.197	+	+
P30086	Phosphatidylethanolamine-binding protein 1	*PEBP1*	21.057	0	32.645	+	+
P00558	Phosphoglycerate kinase 1	*PGK1*	44.614	0	112.890	+	+
P18669	Phosphoglycerate mutase 1	*PGAM1*	28.804	0	23.953	+	+
P55058	Phospholipid transfer protein	*PLTP*	54.739	0	8.454	+	+

Accession ID	Protein	Gene	Mol. weight [kDa]	Q-value[a]	Score[b]	Titanium	Ceramic
P05155	Plasma protease C1 inhibitor	*SERPING1*	55.154	0	39.258	+	+
P00747	Plasminogen	*PLG*	90.568	0	260.020	+	+
Q8NC51	Plasminogen activator inhibitor 1 RNA-binding protein	*SERBP1*	44.965	0	68.499	+	+
P13796	Plastin-2	*LCP1*	70.288	0	57.475	+	+
H3BRU6	Poly(rC)-binding protein 2	*PCBP2*	31.600	0	10.627	-	+
P01833	Polymeric immunoglobulin receptor	*PIGR*	83.283	0	323.310	+	+
J3QS39	Polyubiquitin-B	*UBB*	10.469	0	48.002	+	+
H3BPF6	Prefoldin subunit 5	*PFDN5*	17.047	0	10.715	+	-
P07737	Profilin-1	*PFN1*	15.054	0	211.460	+	+
P12273	Prolactin-inducible protein	*PIP*	16.572	0	323.310	+	+
P04280	Proline-rich peptide II-2	*PRB1*	38.545	0.001	2.416	+	+
Q6MZM9	Proline-rich protein 27	*PRR27*	22.720	0	33.831	+	+
Q16378	Proline-rich protein 4	*PRR4*	16.890	0	66.158	+	+
B1AVU8	Prosaposin	*PSAP*	61.692	0	124.030	+	+
Q15185	Prostaglandin E synthase 3	*PTGES3*	19.155	0	11.038	+	-
Q16651	Prostasin	*PRSS8*	36.431	0	16.339	+	-
Q06323	Proteasome activator complex subunit 1	*PSME1*	28.723	0	6.004	+	-
P25787	Proteasome subunit alpha type-2	*PSMA2*	25.898	0	26.686	+	-
P02760	Protein AMBP	*AMBP*	38.999	0.002	4.514	+	+
P07237	Protein disulfide-isomerase	*P4HB*	57.116	0	228.670	+	+
P30101	Protein disulfide-isomerase A3	*PDIA3*	56.782	0	32.603	+	+
H7BZJ3	Protein disulfide-isomerase A3	*PDIA3*	13.519	0.001	2.7767	+	-
P13667	Protein disulfide-isomerase A4	*PDIA4*	72.932	0	13.580	+	+
B3EWG6	Protein FAM25G	*FAM25G*	9.320	0	50.200	+	+
A0A0A0MS12	Protein IGHV4-34	*IGHV4-34*	13.815	0.003	1.573	+	+
A0A087WZW8	Protein IGKV3-11	*IGKV3-11*	25.600	0	323.310	+	+

Accession ID	Protein	Gene	Mol. weight [kDa]	Q-value[a]	Score[b]	Titanium	Ceramic
P31949	Protein S100-A11	*S100A11*	11.740	0	111.790	+	+
P80511	Protein S100-A12	*S100A12*	10.575	0	74.065	+	+
R4GN49	Protein S100-A2	*S100A2*	7.3243	0.005	1.242	+	+
Q5RHS7	Protein S100-A2	*S100A2*	10.970	0	45.710	+	+
P26447	Protein S100-A4	*S100A4*	11.728	0.002	3.251	-	+
R4GN98	Protein S100-A6	*S100A6*	9.681	0	33.337	+	+
P05109	Protein S100-A8	*S100A8*	10.834	0	180.880	+	+
P06702	Protein S100-A9	*S100A9*	13.242	0	323.310	+	+
P25815	Protein S100-P	*S100P*	10.400	0	6.811	+	+
Q01105	Protein SET	*SET*	33.488	0	9.641	+	+
Q08188	Protein-glutamine gamma-glutamyltransferase E	*TGM3*	76.631	0	156.510	+	+
E9PIT3	Prothrombin	*F2*	65.408	0	183.650	+	+
B8ZZQ6	Prothymosin alpha	*PTMA*	11.758	0	36.058	+	+
P14618	Pyruvate kinase PKM	*PKM*	57.936	0	282.770	+	+
P50395	Rab GDP dissociation inhibitor alpha	*GDI2*	50.663	0	22.307	+	+
C9JJ34	Ran-specific GTPase-activating protein	*RANBP1*	18.762	0	13.345	+	-
B1AH78	Ras-related C3 botulinum toxin substrate 1	*RAC1*	18.502	0	6.809	+	+
P62834	Ras-related protein Rap-1A	*RAP1A*	20.987	0	8.413	+	-
Q9HD89	Resistin	*RETN*	11.419	0.002	3.914	+	-
P52566	Rho GDP-dissociation inhibitor 2	*ARHGDIB*	22.988	0	27.021	+	+
P34096	Ribonuclease 4	*RNASE4*	16.840	0	19.847	+	+
H0YAE9	Ribonuclease T2	*RNASET2*	21.772	0	6.874	+	+
P23921	Ribonucleoside-diphosphate reductase	*RRM1*	90.069	0.001	2.726	+	-
J3QR09	Ribosomal protein L19	*RPL19*	23.134	0.001	1.810	+	-
B1AKD8	Rootletin	*CROCC*	149.060	0	10.328	+	+
P02810	Salivary acidic proline-rich phosphoprotein 1/2	*PRH1*	17.851	0	323.310	+	+

Accession ID	Protein	Gene	Mol. weight [kDa]	Q-value[a]	Score[b]	Titanium	Ceramic
Q8N474	Secreted frizzled-related protein 1	*SFRP1*	35.385	0	9.519	+	+
D6REX5	Selenoprotein P	*SEPP1*	35.116	0	8.370	-	+
Q9NQ38	Serine protease inhibitor Kazal-type 5	*SPINK5*	120.71	0	79.004	+	+
E5RFX6	Serine protease inhibitor Kazal-type 7	*SPINK7*	6.563	0	5.342	-	+
Q9BRF8	Serine/threonine-protein phosphatase CPPED1	*CPPED1*	35.548	0.002	4.828	+	-
P02787	Serotransferrin	*TF*	77.063	0	323.310	+	+
E9PQD6	Serum amyloid A-1 protein	*SAA1*	13.546	0	37.744	+	+
P02743	Serum amyloid P-component	*APCS*	25.387	0	8.186	+	-
Q5T123	SH3 domain-binding glutamic acid-rich-like protein 3	*SH3BGRL3*	9.3804	0	8.192	+	+
P35326	Small proline-rich protein 2A	*SPRR2A*	7.9653	0	59.969	+	+
P35325	Small proline-rich protein 2B	*SPRR2B*	7.9754	0	10.242	+	+
P22532	Small proline-rich protein 2D	*SPRR2D*	7.9053	0.001	2.362	-	+
Q9UBC9	Small proline-rich protein 3	*SPRR3*	18.154	0	323.310	+	+
Q14515	SPARC-like protein 1	*SPARCL1*	75.207	0	323.310	+	+
Q13813	Spectrin alpha chain, non-erythrocytic 1	*SPTAN1*	284.540	0	11.794	+	+
P02808	Statherin	*STATH*	7.304	0	49.528	+	+
P02814	Submaxillary gland androgen-regulated protein 3B	*SMR3B*	8.188	0	47.530	+	+
O00391	Sulfhydryl oxidase 1	*QSOX1*	82.577	0	312.290	+	+
Q6UWP8	Suprabasin	*SBSN*	60.540	0	323.310	+	+
Q9Y490	Talin-1	*TLN1*	269.760	0.002	3.910	+	-
F8VQ14	T-complex protein 1 subunit beta	*CCT2*	44.812	0	28.991	+	-
Q99832	T-complex protein 1 subunit eta	*CCT7*	59.366	0	23.158	+	-
P40227	T-complex protein 1 subunit zeta	*CCT6A*	58.024	0	6.940	+	+
E9PHK0	Tetranectin	*CLEC3B*	17.794	0	31.212	+	+
H7BXY6	Tetraspanin-14	*TSPAN14*	23.918	0.001	1.892	+	-
P10599	Thioredoxin	*TXN*	11.737	0	68.640	+	+

Accession ID	Protein	Gene	Mol. weight [kDa]	Q-value[a]	Score[b]	Titanium	Ceramic
Q9BRA2	Thioredoxin domain-containing protein 17	*TXNDC17*	13.941	0	7.509	+	-
E9PB61	THO complex subunit 4	*ALYREF*	27.557	0	18.031	+	-
C9JGI3	Thymidine phosphorylase	*TYMP*	46.087	0.002	3.263	+	-
P63313	Thymosin beta-10	*TMSB10*	5.026	0	15.236	+	+
P62328	Thymosin beta-4	*TMSB4X*	5.053	0	125.270	+	+
P37837	Transaldolase	*TALDO1*	37.540	0	9.361	+	+
P20061	Transcobalamin-1	*TCN1*	48.206	0	222.500	+	+
X6RJP6	Transgelin-2	*TAGLN2*	21.086	0	109.420	+	+
P55072	Transitional endoplasmic reticulum ATPase	*VCP*	89.321	0	34.183	+	+
P29401	Transketolase	*TKT*	67.877	0	108.970	+	+
J3KPG2	Translationally-controlled tumor protein	*TPT1*	18.108	0.003	1.713	+	-
P02766	Transthyretin	*TTR*	15.887	0	79.078	+	+
Q07654	Trefoil factor 3	*TFF3*	10.181	0	15.845	+	+
P60174	Triosephosphate isomerase	*TPI1*	30.791	0	76.548	+	+
P68363	Tubulin alpha-1B chain	*TUBA1B*	50.151	0	161.450	+	+
Q5JP53	Tubulin beta chain	*TUBB*	47.766	0	285.590	+	-
Q9BW30	Tubulin polymerization-promoting protein family member 3	*TPPP3*	18.985	0	14.834	+	+
E5RIW3	Tubulin-specific chaperone A	*TBCA*	10.080	0	41.274	+	+
P68036	Ubiquitin-conjugating enzyme E2 L3	*UBE2L3*	17.861	0	7.170	+	+
Q6P5S2	UPF0762 protein C6orf58	*C6orf58*	37.926	0	323.310	+	+
P08670	Vimentin	*VIM*	53.651	0	323.310	+	+
D6RF35	Vitamin D-binding protein	*GC*	53.020	0	158.070	+	+
P04004	Vitronectin	*VTN*	54.305	0	105.290	+	+
Q86VR7	V-set and immunoglobulin domain-containing protein 10-like	*VSIG10L*	91.624	0.002	3.246	+	+
Q14508	WAP four-disulfide core domain protein 2	*WFDC2*	12.993	0	43.544	+	+
25311	Zinc-alpha-2-glycoprotein	*AZGP1*	34.258	0	231.860	+	+

Accession ID	Protein	Gene	Mol. weight [kDa]	Q-value[a]	Score[b]	Titanium	Ceramic
Q96DA0	Zymogen granule protein 16 homolog B	*ZG16B*	22.739	0	323.310	+	+

+: detected; -: not detectable.

[a]Minimum false discovery rate generated by MaxQuant

[b]Andromeda scores generated by MaxQuant
